# The PDGF pathway in breast cancer is linked to tumour aggressiveness, triple-negative subtype and early recurrence

**DOI:** 10.1007/s10549-018-4664-7

**Published:** 2018-01-29

**Authors:** Sara Jansson, Kristina Aaltonen, Pär-Ola Bendahl, Anna-Karin Falck, Maria Karlsson, Kristian Pietras, Lisa Rydén

**Affiliations:** 1 Division of Oncology and Pathology (Clinical Sciences), Department of Clinical Sciences Lund, Lund University, Medicon Village 404:C2, Scheelev. 2, SE-223 81 Lund, Sweden; 2 Department of Surgery, Helsingborg Hospital, SE-251 87 Helsingborg, Sweden; 3 Division of Surgery, Department of Clinical Sciences Lund, Skåne University Hospital, Lund University, SE-221 85 Lund, Sweden; 4 Division of Translational Cancer Research, Department of Laboratory Medicine, Medicon Village, SE-223 81 Lund, Sweden; 5 Department of Surgery and Gastroenterology, Skåne University Hospital, SE-205 02 Malmö, Sweden

**Keywords:** Breast cancer, Triple-negative breast cancer, Platelet-derived growth factor receptor, Platelet-derived growth factor-CC, Tyrosine kinase receptor, Targeted therapy

## Abstract

**Purpose:**

The platelet-derived growth factor (PDGF) signalling pathway is often dysregulated in cancer and PDGF-receptor expression has been linked to unfavourable prognostic factors in breast cancer (e.g. ER negativity, high Ki67 and high grade). This study aimed to evaluate the expression of PDGFRα, PDGFRβ and ligand PDGF-CC in breast cancer in relation to molecular subtypes and prognosis.

**Methods:**

Protein expression of tumour and/or stromal cell PDGFRα, PDGFRβ and PDGF-CC was evaluated in primary tumours (*N* = 489), synchronous lymph node metastases (*N* = 135) and asynchronous recurrences (*N* = 39) using immunohistochemistry in a prospectively maintained cohort of primary breast cancer patients included during 1999–2003. Distant recurrence-free interval (DRFi) was the primary end-point.

**Results:**

High expression of all investigated PDGF family members correlated to increasing Nottingham histopathological grade and high Ki67. Tumour cells displayed high expression of PDGFRα in 20%, and PDGF-CC in 21% of primary tumours, which correlated with the triple-negative subtype (TNBC). Patients with high PDGF-CC had inferior prognosis (*P* = 0.04) in terms of 5-year DRFi, whereas PDGFRα was up-regulated in lymph node metastasis and recurrences compared to primary tumours. High primary tumour PDGFRα was associated with increased risk of central nervous system (CNS) recurrence.

**Conclusions:**

High PDGFRα and PDGF-CC expression were linked to breast cancer with an aggressive biological phenotype, e.g. the TNBC subtype, and high PDGF-CC increased the risk of 5-year distant recurrence. Tumour cell PDGFRα was significantly up-regulated in lymph node metastases and asynchronous recurrences. Our findings support an active role of the PDGF signalling pathway in tumour progression.

**Electronic supplementary material:**

The online version of this article (10.1007/s10549-018-4664-7) contains supplementary material, which is available to authorized users.

## Introduction

The platelet-derived growth factor receptor (PDGFR) pathway is a signalling network of importance for the normal development of cells of mesenchymal origin. The PDGF signalling network consists of two tyrosine kinase receptors, PDGFR alpha (PDGFRα) and PDGFR beta (PDGFRβ), and five ligands PDGF-AA, PDGF-BB, PDGF-AB, PDGF-CC and PDGF-DD, which are formed from four gene products (PDGF A, B, C and D) [1]. Autocrine stimulation of the pathway is frequently observed in various neoplasms, such as gliomas [2], gastro-intestinal stromal tumours (GISTs) [3] and chronic myelomonocytic leukaemia [4]. In addition, dysregulation of paracrine PDGFR signalling can cause extracellular matrix remodelling in a tumour-promoting way to facilitate migration, invasion, angiogenesis and possibly also lymph angiogenesis [5, 6].

In breast cancer, most studies of the PDGF signalling pathway examine the expression of PDGFRα and PDGFRβ. PDGFRα expression has been described both in stroma and in tumour cells. High tumour cell PDGFRα expression has been linked to lymph node metastasis, human epidermal growth factor receptor 2 (HER2) positivity [7], high histologic grade, oestrogen receptor (ER) negativity, progesterone receptor (PR) negativity and the triple-negative breast cancer subtype (TNBC) [8, 9]. High stromal PDGFRα has been linked to HER2 positivity and high Ki67 [9]. PDGFRβ expression in breast cancer has only been reported in stroma and high expression has been associated to HER2 positivity, high Ki67 [9], high histopathologic grade, ER negativity and shorter survival [10].

The PDGF-CC ligand was discovered towards the end of the 1990s and it has been shown to be involved in tumour growth by paracrine signalling through PDGFRα in malignant melanoma and cervical carcinoma [11, 12]. However, to date little is known about the role of PDGF-CC in breast cancer. We have recently shown that tumour cell-derived PDGF-CC acts on neighbouring tumour stromal cells in mouse models, and we proposed that the PDGF signalling pathway is a regulator of breast tumour subtype with high PDGF-CC driving breast tumours towards a more basal-like phenotype [13]. Basal-like breast cancer (BLBC) is a subtype of breast cancer defined by gene expression profiling and it is characterised by the expression of genes related to basal epithelial cells such as keratin 5, keratin 17, integrin-β4 and laminin [14]. BLBC largely overlaps with the molecular surrogate breast cancer subtype TNBC, which is defined by immunohistochemistry as being negative for the ER, PR and HER2 receptors [15]. Patients with TNBC are generally younger, have larger tumours with high grade at diagnosis and are more frequently BRCA-mutation carriers [16]. They also have a poor prognosis and no targeted therapies are hitherto available. It is thus particularly desirable to find targetable tumour-driving pathways within TNBC [17]. The PDGF signalling network holds promise of such a pathway, in TNBC in particular and in breast cancer in general.

The aim of the present study was to explore the expression of PDGFRα, PDGFRβ and ligand PDGF-CC in breast cancer to elucidate if these proteins are associated with molecular surrogate subtypes, type of metastatic location and prognosis in breast cancer. A secondary aim was to explore the relation to tumour progression by investigating changes in protein expression between primary tumour, synchronous lymph node metastases and asynchronous recurrences.

## Materials and methods

### Patients

The patient cohort in this study was originally assembled for a prospective observational study with the aim of evaluating the presence and prognostic value of disseminated tumour cells in the bone marrow of patients diagnosed with primary breast cancer [18]. The study was approved by the Lund University ethics committee, and a written informed consent was obtained from all the included patients (LU699-09, LU75-02). Further information about the patient cohort has been published elsewhere [8, 18–20]. Detailed information on routine prognostic factors, St Gallen molecular subtype and clinical follow-up data were assembled for all the patients as described by Falck et al. [19, 20]. The latest review of patient charts to evaluate recurrence status was performed in 2015 and all events until November 2015 were recorded. A recurrence was defined as a radiologic and/or biopsy verified breast cancer-related event. Recurrences within the breast, chest wall, axilla or loco-regional lymph nodes were considered as loco-regional, whereas recurrences in distant organs [e.g. liver, lung and central nervous system (CNS)] were considered as distant. Data on breast cancer-related death were retrieved from the Swedish Register of Causes of Death (Central Statistics Office). We followed the REporting recommendations for tumour MARKer prognostic studies (REMARK) criteria [21].

### Tissue microarray and immunohistochemistry

Tissue microarrays (TMAs) of formalin-fixed, paraffin-embedded tumour tissue samples were retrieved from the Department of Pathology, in Lund and Helsingborg, Sweden. TMAs consisted of tissue core biopsies of 1.0 mm in diameter taken from representative areas of invasive breast cancer using a tissue array machine (TMArrayer™, Pathology Devices, Inc.). Two core biopsies were taken from each tumour sample. TMA sections between 3 and 4 μm thick were transferred to glass slides (Menzel Super frost plus, Thermo Scientific, Germany), dried at room temperature and baked in a heat chamber for 2 h at 60 °C. After deparaffinisation and antigen retrieval, immunohistochemistry (IHC) staining was performed using Autostainer Plus (Dako Denmark A/S, Glostrup, Denmark). The following antibodies and dilutions were used: PDGFRα (#3164 Cell Signaling Technology, Inc., Danvers, MA, USA, 1:100), PDGFRβ (#3169 from Cell Signaling Technology, Inc., Danvers, MA, USA, 1:100) and PDGF-CC (Karolinska Institute, Stockholm, Sweden, 1:2000). A Rabbit Link K8009 (Dako Denmark A/S, Glostrup, Denmark) was used to amplify the signal of the primary PDGFRα antibody. All slides were counterstained with Mayer’s Haematoxylin applied for 2 min and a visualization kit K801021-2 (Dako Denmark A/S, Glostrup, Denmark) was used for all stainings.

PDGFRα was evaluated both in tumour cells and stromal cells, whereas PDGFRβ was only evaluated in stromal cells, as no staining was found in tumour cells, and PDGF-CC only in tumour cells. Tumour cell PDGFRα assessment was performed using a histoscore from 0 to 12 as previously described [8].

Stromal PDGFRα, PDGFRβ and tumour cell PDGF-CC were assessed by a clinical pathologist (DG) and scored for staining intensity 0–3 (0 = negative, 1 = weak, 2 = intermediate and 3 = strong). A tumour was considered positive (high expression) when the intensity was 3 and negative (intermediate, low or absent expression) if the intensity was 0–2 [10].

For both tumour cell and stromal cell staining, only TMA core biopsies with > 100 invasive tumour cells were included. In the statistical analyses, the highest value for the two cores was used. PDGFRα, PDGFRβ and PDGF-CC expression was analysed both as ordinal variables, and dichotomized into positive (high expression) versus negative (intermediate, low or absent expression) as described above. Staining procedures and assessments of ER, PR, HER2, EGFR and CK5/6 have been described in detail elsewhere [19].

### Statistical analysis

The association between biomarker expression and different patient and tumour characteristics was analysed with binary logistic regression. Fisher’s exact test was used to explore any association between primary tumour PDGF expression (positive or negative) and site of recurrence. Comparison of biomarker status between primary tumours, lymph node metastases and distant recurrences was performed using the McNemar test. The Jonckheere Terpstra test was used to test for ordered differences between receptor and ligand status. To evaluate survival effect, Kaplan–Meier survival curves and log rank test or log rank linear trends for factor levels were used. Hazard ratios (HR) were calculated by Cox regression and multivariable analyses were adjusted for age, tumour size, synchronous lymph node metastasis, Nottingham histological grade (NHG) and subtype according to St Gallen 2013. Distant recurrence-free interval (DRFi) was used as end-point. DRFi was defined as the time from surgery of the primary tumour until radiologic and/or biopsy verified distant recurrence or breast cancer-related death. Patients without event were censored at last medical follow-up visit. DRFi was stratified into three time intervals (0–5 years, > 5–10 years and > 10 years) which were analysed separately to explore short- and long-term effects of the biomarkers, respectively. The results from these post hoc analyses should be interpreted cautiously. Statistical calculations were performed using IBM SPSS Statistics (version 24.0, IBM, Armonk, NY, USA). All *P* values presented are two-sided and *P* values < 0.05 were considered statistically significant.

## Results

### Patient and tumour characteristics

**Fig. 1:**
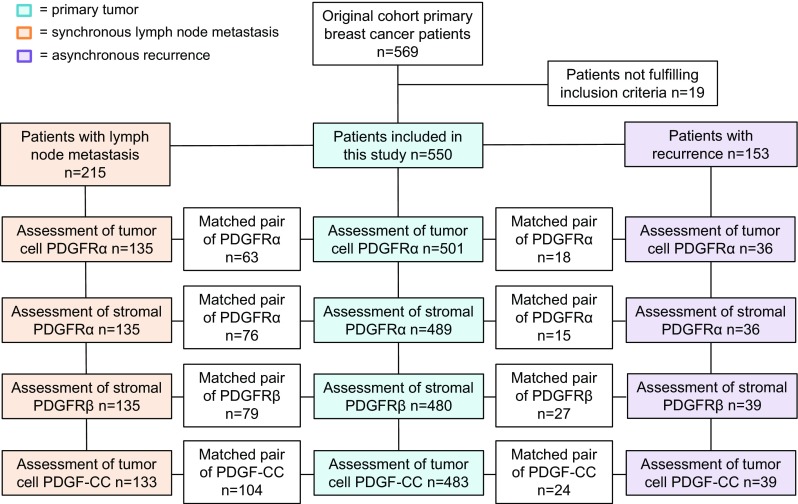
Flowchart of patient cohort and biomarker expression in primary tumour, synchronous lymph node metastases and asynchronous recurrences. Due to limited remaining tissue material, synchronous lymph node metastasis and asynchronous recurrences were only evaluable in 135 and 39 patients, respectively. Boxes are inserted into the flowchart displaying information on matched pairs, i.e. numbers of primary tumours and nodes, and primary tumours and recurrences displaying identical scoring of each marker, respectively (positive–positive or negative–negative)

A total of 550 patients were included in the present study. Primary tumours from 473 patients (86%) had a known St Gallen subtype according to guidelines from 2013 [15] (Luminal A = 193; Luminal B HER2− = 153; Luminal B HER2+ = 79; HER2+ = 15; triple-negative breast cancer [TNBC] = 33). Further data on patient and tumour characteristics are provided in Table 1. Median age at diagnosis was 57.8 years (26–91 years) and median follow-up time for patients alive without any event was 13.7 years (1.0–16.6 years). Synchronous lymph node metastases were present in 215 patients and asynchronous recurrence was recorded in 153 patients. Amongst the asynchronous recurrences, 76 patients had a local, loco-regional or contralateral event and 77 patients developed distant recurrence. Eighty-nine patients experienced early recurrence (within 5 years of primary breast cancer diagnosis). Due to limited remaining tissue material, synchronous lymph node metastasis and asynchronous recurrences were only evaluable in 135 and 39 patients, respectively (Fig. 1).

**Table 1:** Odds ratio (OR) of biomarker expression in relation to tumour and patient characteristics

Biomarker expression	All patients *N* = 550 *N* (%)	PDGFRα in tumour cells OR (95% CI)	PDGFRα in stroma OR (95% CI)	PDGFRβ in stroma OR (95% CI)	PDGF-CC in tumour cells OR (95% CI)
Age (median)					
< 50	110 (20)	1.0	1.0	1.0	1.0
≥ 50	440 (80)	0.80 (0.47–1.35)	0.46 (0.29–0.73)*	0.56 (0.35–0.89)*	0.33 (0.21–0.55)*
T-size					
≤ 20 mm	366 (67)	1.0	1.0	1.0	1.0
> 20 mm	179 (33)	0.86 (0.54–1.38)	0.97 (0.66–1.40)	1.08 (0.71–1.66)	2.14 (1.37–3.35)*
Node status					
N0	319 (60)	1.0	1.0	1.0	1.0
N+	215 (40)	0.67 (0.42–1.06)	1.23 (0.85–1.77)	1.21 (0.80–1.82)	1.10 (0.70–1.71)
NHG					
I	118 (22)	1.0	1.0	1.0	1.0
II	287 (54)	0.99 (0.54–1.80)	1.08 (0.68–1.72)	1.41 (0.80–2.51)	1.26 (0.63–2.54)
III	129 (24)	1.94 (1.03–3.69)*	3.01 (1.74–5.21)*	2.55 (1.37–4.73)*	7.72 (2.83–11.52)*
Ki67					
Low (≤ 20)	335 (67)	1.0	1.0	1.0	1.0
High (> 20)	165 (33)	3.47 (2.20–5.48)*	1.82 (1.23–2.67)*	2.12 (1.39–3.23)*	5.13 (3.21–8.20)*
St Gallen subtype					
Luminal A	193 (41)	1.0	1.0	1.0	1.0
Luminal B HER2−	153 (32)	1.74 (1.01–3.00)*	1.07 (0.70–1.66)	1.03 (0.63–1.70)	3.60 (1.84–7.04)*
Luminal B HER2+	79 (17)	1.38 (0.70–2.71)	1.58 (0.92–2.71)	1.40 (0.78–2.52)	3.34 (1.55–7.19)*
HER2-type	15 (3)	0.90 (0.19–4.25)	6.68 (1.44–30.97)*	0.84 (0.22–3.19)	10.10 (2.99–34.19)*
TNBC	33 (7)	2.71 (1.19–6.18)*	1.65 (0.78–3.48)	0.94 (0.39–2.23)	30.12 (11.72–77.43)*
ER					
Neg	57 (11)	1.0	1.0	1.0	1.0
Pos	449 (89)	0.82 (0.42–1.58)	0.41 (0.22–0.75)*	1.06 (0.56–2.03)	0.10 (0.06–0.19)*
PR					
Neg	108 (23)	1.0	1.0	1.0	1.0
Pos	367 (77)	0.77 (0.46–1.30)	0.75 (0.49–1.16)	0.93 (0.57–1.51)	0.18 (0.11–0.29)*
EGFR					
Neg	419 (83)	1.0	1.0	1.0	1.0
Pos	84 (17)	1.25 (0.71–2.21)	1.98 (1.21–3.22)*	1.49 (0.89–2.49)	5.97 (3.56–10.01)*
CK5/6					
Neg	378 (75)	1.0	1.0	1.0	1.0
Pos	124 (25)	1.65 (1.02–2.68)*	1.31 (0.87–2.00)	1.00 (0.62–1.58)	3.67 (2.30–5.87)*
HER2					
Neg	440 (88)	1.0	1.0	1.0	1.0
Pos	62 (12)	0.86 (0.49–1.51)	1.90 (1.20–3.00)*	1.42 (0.88–2.32)	1.52 (0.91–2.53)
Recurrence					
No	398 (72)	1.0	1.0	1.0	1.0
Loco-regional or contralateral	75 (14)	1.22 (0.65–2.31)	1.80 (1.03–3.16)*	1.10 (0.61–1.96)	2.19 (1.23–3.91)*
Distant	77 (14)	1.08 (0.58–2.02)	0.76 (0.40–1.46)	0.82 (0.45–1.50)	1.01 (0.53–1.92)
Metastatic location					
Bone	28 (36)	1.0	1.0	1.0	1.0
Visceral	43 (56)	1.22 (0.32–4.65)	0.52 (0.19–1.42)	0.84 (0.27–2.64)	0.74 (0.20–2.74)
CNS	6 (8)	11.50 (1.55–85.15)*	0.54 (0.08–3.45)	0	4.2 (0.65–27.36)

*NHG* Nottingham histological grade, *ER* oestrogen receptor, *PR* progesterone receptor, *EGFR* epidermal growth factor receptor; *CK5/6* cytokeratin 5/6, *HER2* human epidermal growth factor receptor 2, *TNBC* triple-negative breast cancer, *CNS* central nervous system

*Significant at the 0.05 level

### Patient and tumour characteristics in relation to primary tumour expression of PDGFRα, PDGFRβ and PDGF-CC

**Fig. 2:**
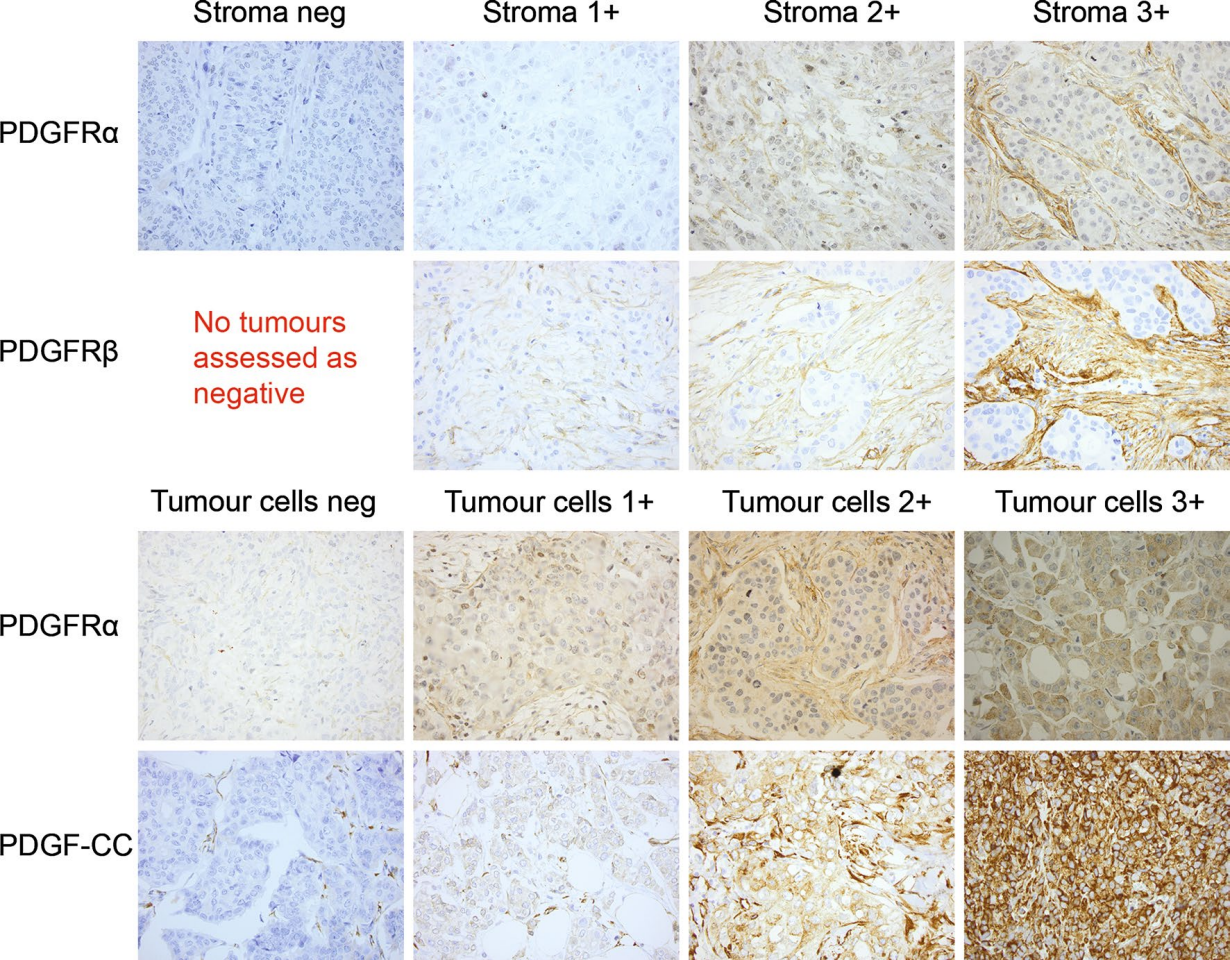
Examples of IHC stainings for the members of the PDGF family. PDGFRα in stromal cells (1st row), PDGFRβ in stromal cells (2nd row), PDGFRα in tumour cells (3rd row) and PDGF-CC in tumour cells (4th row). Original magnification ×40

Examples of IHC staining are given in Fig. 2. Tumour cells showed high expression of PDGFRα in 100 (20%) and PDGF-CC in 103 (21%) of the evaluated primary tumours. Stromal cells showed high expression of PDGFRα in 243 (50%) and PDGFRβ in 128 (27%) of the evaluated primary tumours. High expression of all investigated PDGF family members correlated to increasing NHG and high Ki67, and also to different degrees to TNBC, expression of cytokeratin 5/6 (CK5/6+), young age (< 50 years), large tumour size, ER−, PR− and EGFR+ (Table 1).

**Fig. 3:**
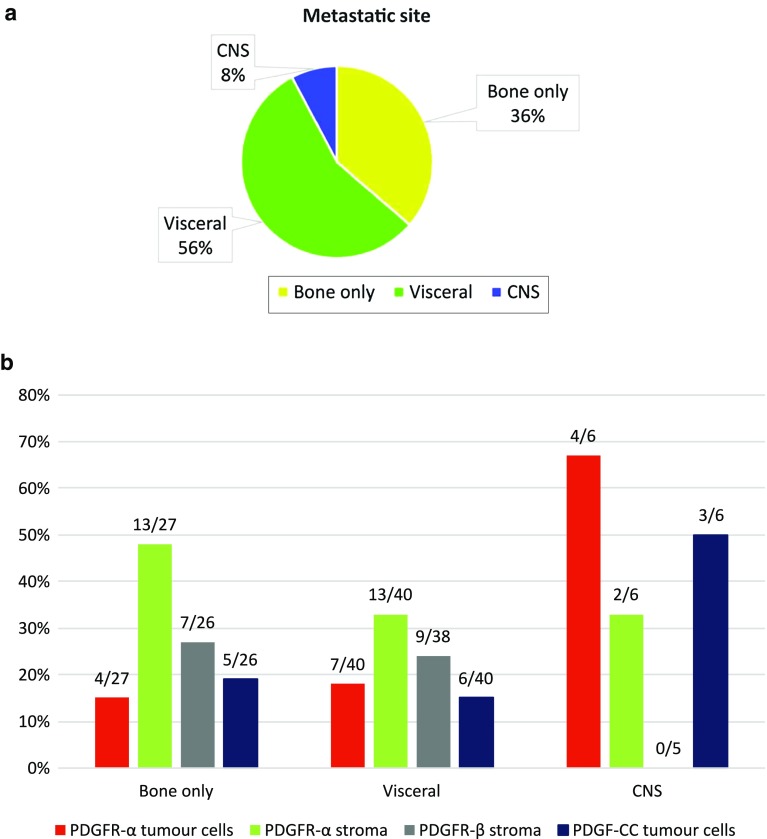
**a** Overview of primary metastatic site at time of recurrence. **b** Relation between primary tumour PDGF expression (receptors α, β or ligand –CC) and site of distant recurrence

The 77 patients that developed distant recurrence were divided into three groups based on the primary distant metastatic site; bone only (28 patients), visceral (43 patients) and CNS (6 patients) (Fig. 3a). If several primary metastatic locations were present, the worst was recorded in the following order: 1: CNS, 2: visceral and 3: bone. The relation between primary tumour PDGF expression and site of distant recurrence is given in Fig. 3b. Recurrence within CNS was more common in patients with high primary tumour cell PDGFRα expression, 4/15 (27%) versus 2/58 (3%), *P* = 0.01.

### Biomarker expression and tumour progression

Figure 1 presents an overview of the PDGF assessments at different locations. Tumour cells showed high expression of PDGFRα in 76 (56%) and PDGF-CC in 29 (22%) of synchronous lymph node metastasis, whereas stromal cells showed high expression of PDGFRα in 68 (50%) and PDGFRβ in 46 (34%). In asynchronous recurrences, tumour cells showed high expression of PDGFRα in 23 (64%) and PDGF-CC in 8 (21%). Stromal cells showed high expression of PDGFRα in 11 (31%) and PDGFRβ in 2 (5%) of evaluated asynchronous recurrences.

A substantial number of tumours displayed a shift in biomarker expression from primary tumour to lymph node metastases and recurrences. The shift was significantly skewed for PDGFRα expression in tumour cells, which was up-regulated in lymph node metastases and recurrences, and for stromal PDGFRβ expression, which was down-regulated in recurrences (Table 2).

**Table 2:** Biomarker concordance and discordance in matched pairs of primary tumours versus corresponding lymph node metastases and asynchronous recurrences

Biomarker expression	PDGFRα in tumour cells		PDGFRα in stroma		PDGFRβ in stroma		PDGF-CC in tumour cells	
Location	*N* (%)	P*	*N* (%)	P*	*N* (%)	P*	*N* (%)	P*
PT versus *N*								
PT pos/N pos	11 (8)	< 0.001	41 (32)	0.7	17 (14)	0.4	18 (14)	0.2
PT pos/N neg	6 (5)		29 (22)		20 (26)		16 (13)	
PT neg/N pos	61 (47)		25 (19)		27 (21)		8 (6)	
PT neg/N neg	52 (40)		35 (27)		62 (49)		86 (67)	
Total	130 (100)		130 (100)		126 (100)		128 (100)	
PT versus R								
PT pos/R pos	9 (26)	0.02	2 (6)	0.6	0 (0)	0.02	6 (17)	0.07
PT pos/R neg	3 (9)		11 (32)		9 (24)		9 (26)	
PT neg/R pos	13 (38)		8 (24)		1 (3)		2 (6)	
PT neg/R neg	9 (26)		13 (38)		27 (73)		18 (51)	
Total	34 (100)		34 (100)		37 (100)		35 (100)	

*PT* primary tumour, *N* lymph node metastasis, *R* recurrence, *neg* negative, *pos* positive

*McNemar test

### Concomitant expression of ligand PDGF-CC and the PDGF receptors

In total, 80 primary tumours (18%) had concomitant high expression of ligand PDGF-CC in tumour cells and at least one of the PDGF receptors in tumour and/or stromal cells. Forty-one (8%) of primary tumours displayed a high expression of both PDGFRα in tumour cells and PDGF-CC, 63 (13%) of PDGFRα in stromal cells and PDGF-CC, and 36 (7%) of PDGFRβ in stromal cells and PDGF-CC. Only 2 tumours had high expression of PDGFRβ and PDGF-CC in combination with absent or low expression of PDGFRα (in both stromal and tumour cells), whereas 42 tumours had high expression of PDGFRα (in stromal and/or tumour cells) and PDGF-CC in combination with low PDGFRβ (Supplementary Table S1). Forty-nine (10%) of primary tumours had high expression of PDGFRα in both stromal and tumour cells, whereas 182 (37%) had low or absent expression of this receptor in both stromal and tumour cells.

To further explore the relation between PDGF-CC and the PDGF receptors, we analysed receptor–ligand combinations using non-dichotomized data. This showed a significant association between increasing expression of both PDGFRα and PDGFRβ, and increasing PDGF-CC levels (*P* < 0.001 for tumour and stromal cell PDGFRα expression, *P* = 0.004 for stromal cell PDGFRβ expression, Supplementary Fig. S1a–c.).

All combinations of concomitant high expression of ligand PDGF-CC and either of the PDGF receptors were significantly associated with young age, increasing NHG, high Ki67, TNBC, ER−, PR−, CK5/6+ and EGFR+ (Supplementary Table S2). No significant difference was seen in survival for patients carrying tumours with concomitant high receptor and ligand expression (data not shown).

### Primary tumour biomarker expression and prognosis

**Fig. 4:**
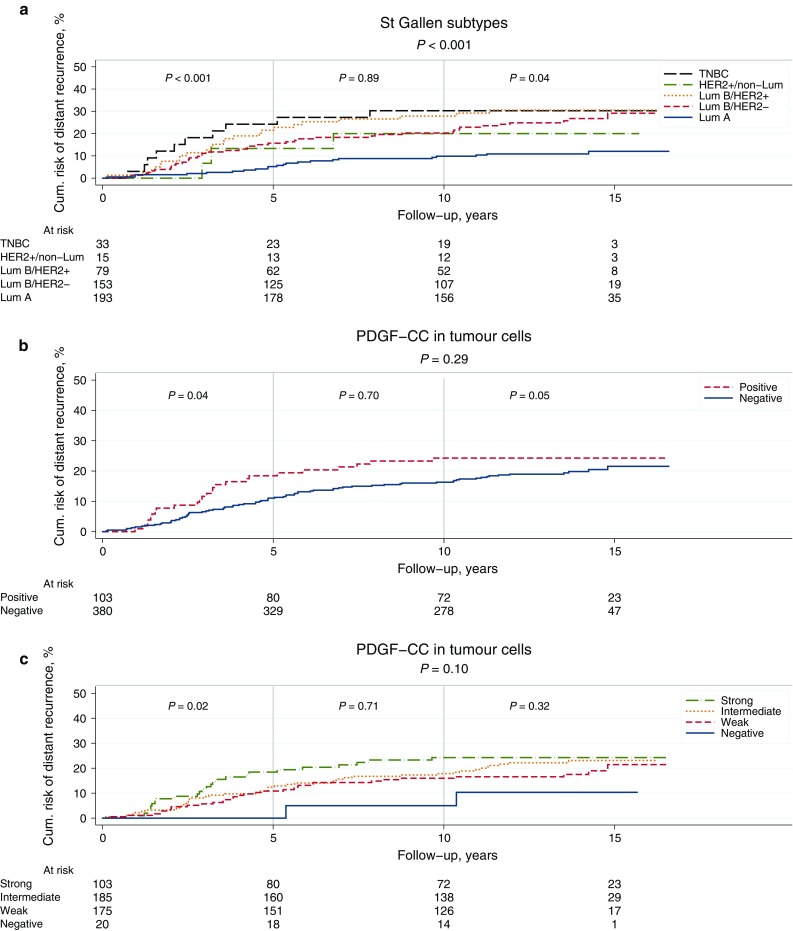
**a**–**c** Kaplan–Meier survival curves showing DRFi (years) in relation to St Gallen molecular subtypes (**a**), expression of PDGF-CC in tumour cells dichotomized into positive versus negative (**b**), and staining intensity of PDGF-CC ranging from 0 (negative) to 3 (strong) (**c**). *P* values from log rank test and log rank linear trends for factor levels

One hundred and eleven patients (20%) experienced distant breast cancer recurrence and/or breast cancer-related death and contributed to events in DRFi. Kaplan–Meier and log rank test showed a significant difference in DRFi over different breast cancer subtypes (Fig. 4a, *P* < 0.001). As expected, Luminal A had the best prognosis and TNBC the worst.

For patients with primary tumours positive versus negative for PDGF-CC, no significant difference in DRFi was found over the complete follow-up time (Fig. 4b, *P* = 0.30). However, the survival curve indicated a prognostic effect over the first few years following breast cancer diagnosis. We thus divided the follow-up time into three intervals, 0–5 years, > 5–10 years and > 10 years. This revealed a significant increased risk of early breast cancer event (recurrence or breast cancer-related death within 5 years of primary diagnosis) in the group of patients with tumours positive for PDGF-CC [Fig. 4b, HR 1.77 (95% confidence interval (CI) 1.03–3.04), *P* = 0.04]. This increase did not remain significant in multivariable analysis adjusted for age, tumour size, node status, NHG and St Gallen molecular subtype (HR 1.14; 95% CI 0.59–2.19) (Supplementary Table S3). For the late events (occurring after 10 years), there was an increased risk amongst patients with tumours negative for PDGF-CC.

We also analysed the intensity of PDGF-CC expression (0–3) and found a trend towards a worse prognosis with increasing expression of PDGF-CC over the whole time interval and a significantly worse prognosis during the first 5 years (Fig. 4c, log rank linear trends for factor levels *P* = 0.1 and *P* = 0.02, respectively).

Analysis of DRFi in relation to PDGF-receptor expression showed no difference in prognosis for patients with tumours positive versus negative for PDGFRα or PDGFRβ over the whole follow-up period (Supplementary Fig. S2a–c).

## Discussion

We present novel data on expression of PDGFRα, PDGFRβ and ligand PDGF-CC in primary breast tumours, synchronous lymph node metastasis and asynchronous recurrences in relation to St Gallen Molecular subtypes and long-term follow-up data in a large cohort of primary breast cancer patients. Data are accumulating on the importance of the PDGF receptors α and β in breast cancer, but the presented results have been conflicting and not related to modern pathology. Furthermore, little is known concerning the role of ligand PDGF-CC. We found that high expression of the investigated members of the PDGF family correlated to several prognostic patient and tumour characteristics that indicate tumours’ inherent biological aggressiveness (e.g. younger age, increasing NHG, high Ki67 and negative ER and PR). This is in line with what has been reported in some previous studies [9, 10] but it contrasts with Weigel et al. who found no significant correlations between PDGFR and the expression of established prognostic biomarkers. Reasons for diverging data could be the use of different antibodies, different inclusion criteria and the location of marker expression.

Interestingly, we report for the first time a significant up-regulation of tumour cell PDGFRα expression in lymph node metastasis and asynchronous recurrences, as a sign of its role in tumour progression. Moreover, we found a significantly increased risk of early breast cancer recurrence amongst patients with tumours expressing increasing levels of PDGF-CC. This effect was visualised by the Kaplan–Meier survival curves which indicate that the prognosis is related to the level of PDGF-CC expression (Fig. 4c). Up-regulation of members of the PDGF signalling pathway has previously been shown to occur during epithelial-to-mesenchymal transition in in vitro/in vivo cellular models [22], and subsequent experiments in mouse tumour models present support that an autocrine PDGF/PDGFR loop contribute to tumour progression and metastasis in vivo [23]. These studies conclude that PDGFR signalling plays an essential role during cancer progression, as has also been observed in our findings.

Approximately 30% of women with primary breast cancer eventually experience distant breast cancer recurrence. We found that high expression of PDGFRα in tumour cells of the primary tumour was correlated to subsequent first distant recurrence occurring within CNS. Even if prognosis in metastatic breast cancer has improved over the past decades [24], metastasis within CNS is a major limitation of life quality and survival [25]. Deciphering the biology behind metastasis and target organs for metastatic spread is important in order to develop targeted therapies. In glioblastoma, autocrine signalling by PDGF-CC/PDGFRα has been proposed to have a role in tumour development and Lokker et al. detected concomitant expression of PDGF-CC and PDGFRα in 6/6 tested glioblastoma cell lines, and 5/5 investigated primary glioblastoma tissue samples [26]. Furthermore, PDGF-CC has been shown to increase the permeability of the blood–brain barrier (BBB) [27], and the role of the PDGFRα/PDGF-CC pathway relative to BBB dysfunction in neurological disorders was recently reviewed [28]. In our cohort, patients who developed CNS metastases had primary tumours with high expression of tumour cell PDGFRα in 67%, PDGF-CC in 50% and concomitant high ligand/receptor expression in 33% of cases, indicating an active role of the PDGF pathway in these tumours. Thus it is possible that the PDGF pathway is involved when a tumour cell crosses the BBB. The number of patients with CNS recurrence is small within this cohort but the finding is intriguing. Unfortunately, we had no tumour tissue available from CNS metastases to explore the expression of the PDGF members. However, Kim et al. recently investigated the expression of tumour cell PDGFRα in breast cancer CNS metastases and found high expression in 12/38 (32%) metastases [29]. Unfortunately, comparisons between these different studies are limited due to different antibodies and scoring systems for PDGFRα staining.

In this study we also explored concomitant expression of PDGFRα and PDGFRβ, and ligand PDGF-CC. Experiments in vitro have shown that PDGF-CC binds to and stimulates homodimers of PDGFRα, and heterodimers of PDGFRα and PDGFRβ, but not homodimers of PDGFRβ [30]. However, the binding capacity of PDGF-CC in vivo is not completely known and we thus explored all the three receptor/ligand combinations. Almost all tumours with high stromal cell PDGFRβ and high tumour cell PDGF-CC also had high PDGFRα, either in stromal or tumour cells. In contrast, more than half of the tumours with high PDGF-CC and high PDGFRα expression in tumour or stromal cells displayed low PDGFRβ. These findings support that PDGF-CC binds to and signals though receptor combinations including the PDGFRα in vivo. Concomitant PDGFRα and PDGF-CC expression varied markedly over the molecular subtypes where the TNBC displayed co-expression in 59% of the tumours, whereas the luminal subtypes only displayed co-expression in 5% (Luminal A) to 19% (Luminal B HER2+). This adds further evidence to the involvement of the PDGF signalling pathway in TNBC as indicated in previous studies [8, 9, 13] and this pathway might thus be a target for therapy in this difficult-to-treat subgroup of breast cancer.

The strength of the present study is that it is performed in a large cohort of breast cancer patients with long follow-up time (13.7 years). The study enables analysis of the PDGF signalling pathway in tumour tissue biopsies from several locations where data on clinically important biomarkers and St Gallen molecular subtypes were available, which allows to evaluate the study results in relation to modern pathology. A limitation is that there was limited tissue material available from lymph node metastasis and recurrences. Also, we recorded distant recurrence and/or breast cancer-related death in only 20% of patients during the follow-up time diminishing the power of the study.

In conclusion, we show that high expression of PDGFRα, PDGFRβ and ligand PDGF-CC is associated to several important prognostic patient and tumour characteristics in breast cancer, indicating a link to tumours with inherent biological aggressiveness. Tumour cell expression of PDGFRα is commonly up-regulated in lymph node metastases and asynchronous recurrences, whereas high expression of PDGF-CC is related to early breast cancer recurrence supporting an active role of the PDGF signalling pathway in tumour progression. Furthermore, our results indicate an intriguing connection between the PDGF pathway and metastatic spread to the CNS, which merits further exploration. In summary, our data encourage further evaluation of the PDGF receptors and ligands in breast cancer, as well as of strategies to target this pathway since evidence is compiling for its involvement in breast cancer progression.

## Electronic supplementary material

Below is the link to the electronic supplementary material.

Supplementary material 1 (PDF 134 kb)
