# Supplementary material for: The PDGF pathway in breast cancer is linked to tumour aggressiveness, triple-negative subtype and early recurrence
Source: Breast Cancer Res Treat. 2018 Jan 29;169(2):231–41. doi: 10.1007/s10549-018-4664-7 (PMC5945746; doi:10.1007/s10549-018-4664-7)

SUPPLEMENTARY FIGURES AND TABLES

Article title: The PDGF pathway in breast cancer is linked to tumour aggressiveness, triple-negative subtype and early recurrence

Journal name: Breast Cancer Research and Treatment

Authors: Sara Jansson*, Kristina Aaltonen, Pär-Ola Bendahl, Anna-Karin Falck, Maria Karlsson, Kristian Pietras and Lisa Rydén

***Corresponding author:** Sara Jansson, email: sara.jansson@med.lu.se, affiliation: Lund University, Division of Oncology and Pathology, Department of Clinical Sciences Lund, Medicon Village, SE-223 81 Lund, Sweden

Table S1 Correlations between expression of ligand PDGF-CC and PDGF-receptors α and β

		PDGFRα in tumour cells	PDGFRα in stroma	PDGFRβ in stroma	PDGF-CC in tumour cells
PDGFRα in tumour cells	Pearson Correlation	1	-.010	.077	.263**
	Sig. (2-tailed)		.820	.094	.000
	N	501	482	474	473
PDGFRα in stroma	Pearson Correlation		1	.200**	.142**
	Sig. (2-tailed)			.000	.002
	N		489	468	466
PDGFRβ in stroma	Pearson Correlation			1	.116*
	Sig. (2-tailed)				.013
	N			480	463
PDGF-CC in tumour cells	Pearson Correlation				1
	Sig. (2-tailed)				
	N				483
**. Correlation is significant at the 0.01 level (2-tailed).					
*. Correlation is significant at the 0.05 level (2-tailed).					

Table S2 Odds ratio (OR) of concomitant ligand and receptor expression in relation to tumour and patient characteristics

**Biomarker expression**	**All patients N=550**	**PDGFRα in tumour cells + PDGF-CC in tumour cells**	**PDGFRα in stroma + PDGF-CC in tumour cells**	**PDGFRβ in stroma + PDGF-CC in tumour cells**
	N (%)	OR (95% CI)	OR (95% CI)	OR (95% CI)
**Age (median)**				
<50	110 (20)	1.0	1.0	1.0
≥50	440 (80)	**0.35 (0.18-0.69)**	**0.27 (0.15-0.47)**	**0.22 (0.11-0.43)**
**T-size**				
≤20 mm	366 (67)	1.0	1.0	1.0
>20 mm	179 (33)	NS	**1.96 (1.15-3.35)**	NS
**Node status**				
N0	319 (60)	1.0	1.0	1.0
N+	215 (40)	NS	NS	NS
**NHG**				
I	118 (22)	1.0	1.0	1.0
II	287 (54)	NS	NS	NS
III	129 (24)	**5.3 (1.78-15.97)**	**6.83 (2.91-16.01)**	**7.82 (2.28-26.86)**
**Ki67**				
Low (≤20)	335 (67)	1.0	1.0	1.0
High (>20)	165 (33)	**6.18 (3.0-12-73)**	**7.66 (4.17-14.07)**	**11.78 (4.78-29.06)**
**St Gallen subtype**				
Luminal A	193 (41)	1.0	1.0	1.0
Luminal B HER2-	153 (32)	NS	**4.2 (1.62-10.86)**	**6.50 (1.83-23.07)**
Luminal B HER2+	79 (17)	NS	**6.06 (2.21-16.61)**	**5.95 (1.50-23.65)**
HER2-type	15 (3)	NS	**25.57 (6.56-99.67)**	**10.06 (1.53-66.04)**
TNBC	33 (7)	**9.84 (3.53-27.44)**	**20.41 (6.96-59.91)**	**16.89 (4.10-69.59)**
**ER**				
Neg	57 (11)	1.0	1.0	1.0

Pos	449 (89)	**0.30 (0.14-0.64)**	**0.11 (0.06-0.21)**	**0.25 (0.12-0.54)**
**PR**				
Neg	108 (23)	1.0	1.0	1.0
Pos	367 (77)	**0.29 (0.15-0.56)**	**0.19 (0.10-0.33)**	**0.22 (0.11-0.45)**
**EGFR**				
Neg	419 (83)	1.0	1.0	1.0
Pos	84 (17)	**4.3 (2.18-8.48)**	**7.30 (4.09-13.02)**	**5.56 (2.73-11.36)**
**CK5/6**				
Neg	378 (75)	1.0	1.0	1.0
Pos	124 (25)	**4.25 (2.20-8.24)**	**3.11 (1.79-5.39)**	**2.68 (1.33-5.40)**
**HER2**				
Neg	440 (88)	1.0	1.0	1.0
Pos	62 (12)	NS	**2.72 (1.50-4.94)**	NS

NS = not significant.

Table S3 Cox univariable and multivariable regression analysis to evaluate the survival effect of PDGF-CC expression in terms of 5-year DRFi, adjusted for important clinicopathological factors

	**Univariable**		**Multivariable**	
	HR	*P*	HR	*P*
**Age (per year)**				
<50 years	1.00		1.00	
≥50 years	0.79 (0.45-1.38)	0.40	0.95 (0.50-1.78)	0.86
**Tumour size**				
<20 mm	1.00		1.00	
≥20 mm	3.96 (2.40-6.53)	<0.001	2.52 (1.40-4.54)	0.002
**Node**				
N0	1.00		1.00	
N+	4.28 (2.48-7.37)	<0.001	3.15 (1.70-5.84)	<0.001
**NHG**				
I	1.00		1.00	
II	3.40 (1.20-9.62)	0.02	3.80 (0.89-16.28)	0.07
III	7.72 (2.64-21.39)	<0.001	5.14 (1.15-22.96)	0.03
**St Gallen subtype**				
Luminal A	1.00		1.00	
Luminal B HER2-	3.23 (1.54-6.75)	0.002	2.15 (0.97-4.77)	0.06
Luminal B HER2+	4.49 (2.06-9.81)	<0.001	2.52 (1.07-5.93)	0.03
HER2-Type	2.61 (0.57-11.93)	0.22	0.58 (0.07-4.87)	0.61
TNBC	5.57 (2.20-14.11)	<0.001	2.57 (0.83-7.98)	0.10
**PDGF-CC**				
Negative	1.00		1.00	
Positive	1.77 (1.03-3.04)	0.04	1.14 (0.59-2.19)	0.70

Fig. S1a-c Relation between expression levels of the PDGF-receptors and of ligand PDGF-CC.

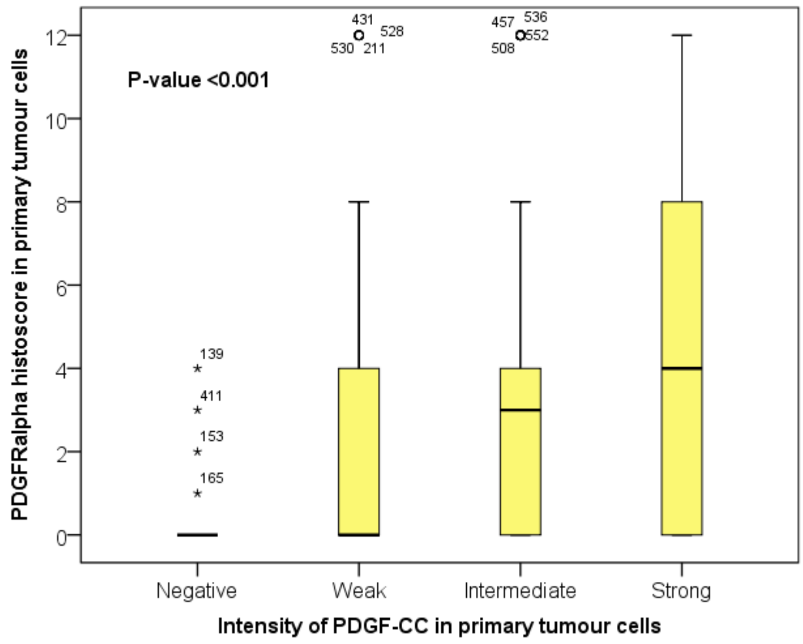

Fig. S1a PDGFRα in tumour cells *vs* PDGF-CC. *P*-value from Jonckheere Terpstra test

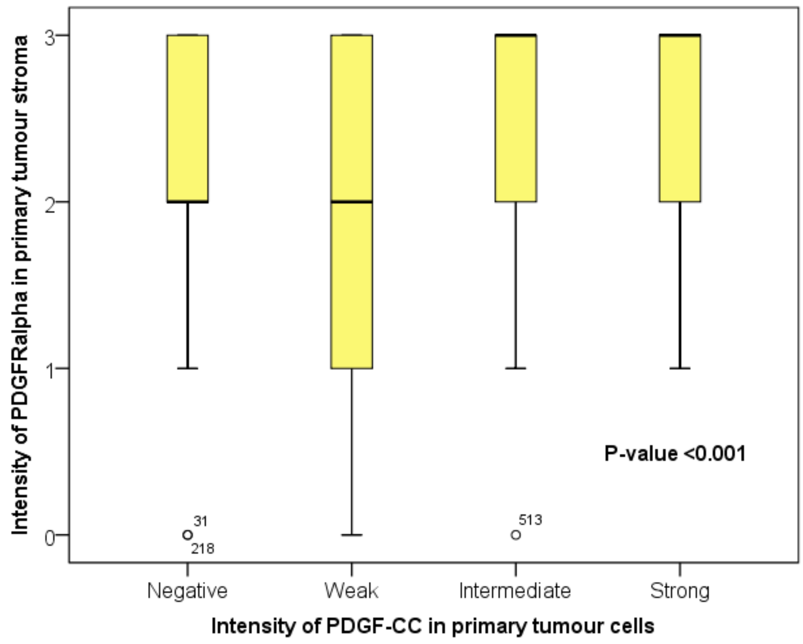

Fig. S1b PDGFRα in tumour stroma *vs* PDGF-CC. *P*-value from Jonckheere Terpstra test

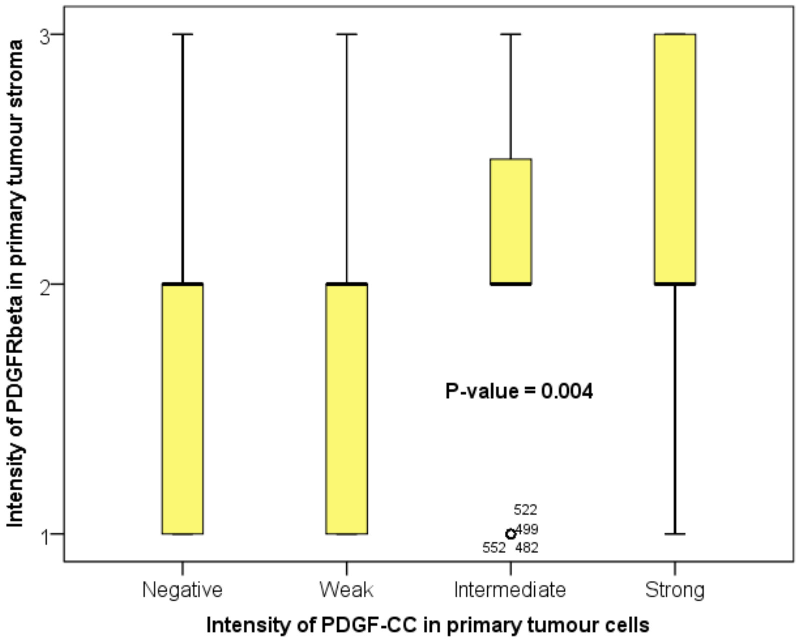

Fig. S1c PDGFRβ in tumour stroma *vs* PDGF-CC. *P*-value from Jonckheere Terpstra test

Fig. S2a-c Kaplan-Meier survival curves showing DRFi (years) in relation to biomarker expression. P-values from log rank test

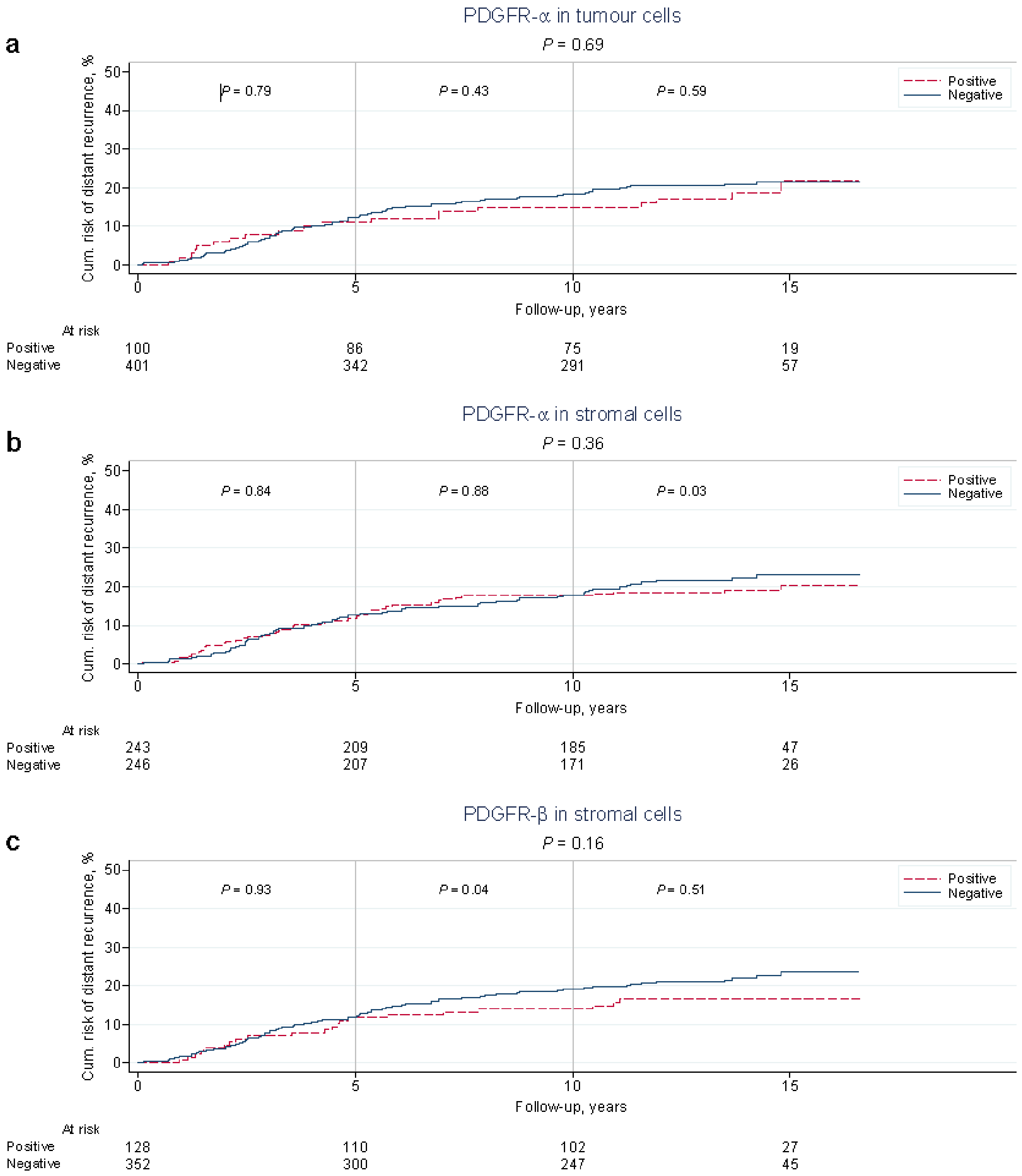

Supplement: Supplementary file 1 — Supplementary material 1 (PDF 134 kb) [file 10549_2018_4664_MOESM1_ESM.pdf]
